# Juvenile pemphigus vulgaris: Literature review and a rare case report

**DOI:** 10.1002/ccr3.8954

**Published:** 2024-05-15

**Authors:** Shyamkumar Sriram, Shamimul Hasan, Shahnaz Mansoori, Shazina Saeed, Abhishek Banerjee, Karthikeyan Ramalingam

**Affiliations:** ^1^ Department of Social and Public Health Ohio University Athens Ohio USA; ^2^ Department of Oral Medicine and Radiology Faculty of Dentistry, Jamia Millia Islamia New Delhi India; ^3^ Amity Institute of Public Health & Hospital Administration Amity University Noida Uttar Pradesh India; ^4^ Oral and Maxillofacial Pathology Awadh Dental College and Hospital Jamshedpur Jharkhand India; ^5^ Department of Oral Pathology and Microbiology, Saveetha Dental College and Hospitals, Saveetha Institute of Medical and Technical Sciences Saveetha University Chennai India

**Keywords:** autoimmune, histopathology, juvenile vulgaris, pemphigus, vesiculobullous disease

## Abstract

Pemphigus vulgaris (PV) is a chronic autoimmune blistering disorder characterized by the loss of intraepithelial adhesion affecting the skin and mucous membranes, predominantly affects females in their fifth and sixth decades of life. Due to its rare occurrence in children and adolescents, there is often a delay in diagnosis and treatment in this age group. PV should always be considered in the differential diagnosis of oral ulcerative and vesiculobullous lesions in both children and adolescents.

## 1 INTRODUCTION

Pemphigus refers to a diverse range of chronic blistering conditions that affect both mucous membranes and skin. These disorders are typified by IgG autoantibodies targeting keratinocyte adhesion proteins (desmogleins Dsg1 and Dsg3). The binding of IgG autoantibodies to desmosomal complexes leads to a disruption in intraepidermal adhesion, which causes loss of cell–cell adhesion (acantholysis). This results in the formation of vesicles, blisters, and erosions on the skin and mucous membranes.^1^, ^2^ Pemphigus vulgaris (PV) is recognized as the most frequently occurring type of pemphigus, accounting for approximately 70% of all cases.^3^

Although PV is considered an autoimmune disorder, the specific mechanism of desmosome breakdown after autoantibody binding remains unclear. Multiple theories, such as the steric hindrance theory, desmoglein compensation theory, multiple hits hypothesis, and antibody‐induced apoptosis and signaling theory, have been proposed in the literature, but have not yielded conclusive results.^4^

Various antigenic triggering factors have also been identified that play a role in PV pathogenesis. These include viral infections, genetic factors, thiol group drugs (penicillamine, captopril, and rifampicin), food (such as garlic), vaccines, radiation therapy, pregnancy, micronutrients, and stress.^5^, ^6^

The worldwide incidence of PV is 0.1–0.5 per 100,000 people per year, however, this varies from 0.17/million/year in France to 6.8/million/year in the United Kingdom. However, the PV incidence in India ranges from 0.09% to 1.8%.^7^ PV is more common in the Jewish populations, particularly those of Ashkenazi origin, and in the Mediterranean.^5^, ^7^, ^8^

PV exhibits an age and site predilection and typically affects females during their fifth or sixth decade of life.^8^ Approximately 1.4% to 3.7% of all PV cases are observed in individuals ≤18 years of age. PV in the pediatric group can be categorized as childhood/pediatric PV, affecting those under 12 years of age, and juvenile/adolescent PV, affecting individuals between the ages of 12 and 18 years. The majority of the pediatric pemphigus cases are of the vulgaris type, generally manifesting at approximately 12 years of age.^9^

Pediatric PV cases can pose a diagnostic challenge because of their rarity, and are frequently identified only after a more advanced clinical presentation.^10^

## 2 CASE PRESENTATION

A 16‐year‐old immunocompetent female patient was referred by a public health camp to our Outpatient Department for evaluation of oral soreness and burning sensations for the last 8 months. She also complained of difficulty in chewing and swallowing.

### 2.1 History and clinical examination

**FIGURE 1:**
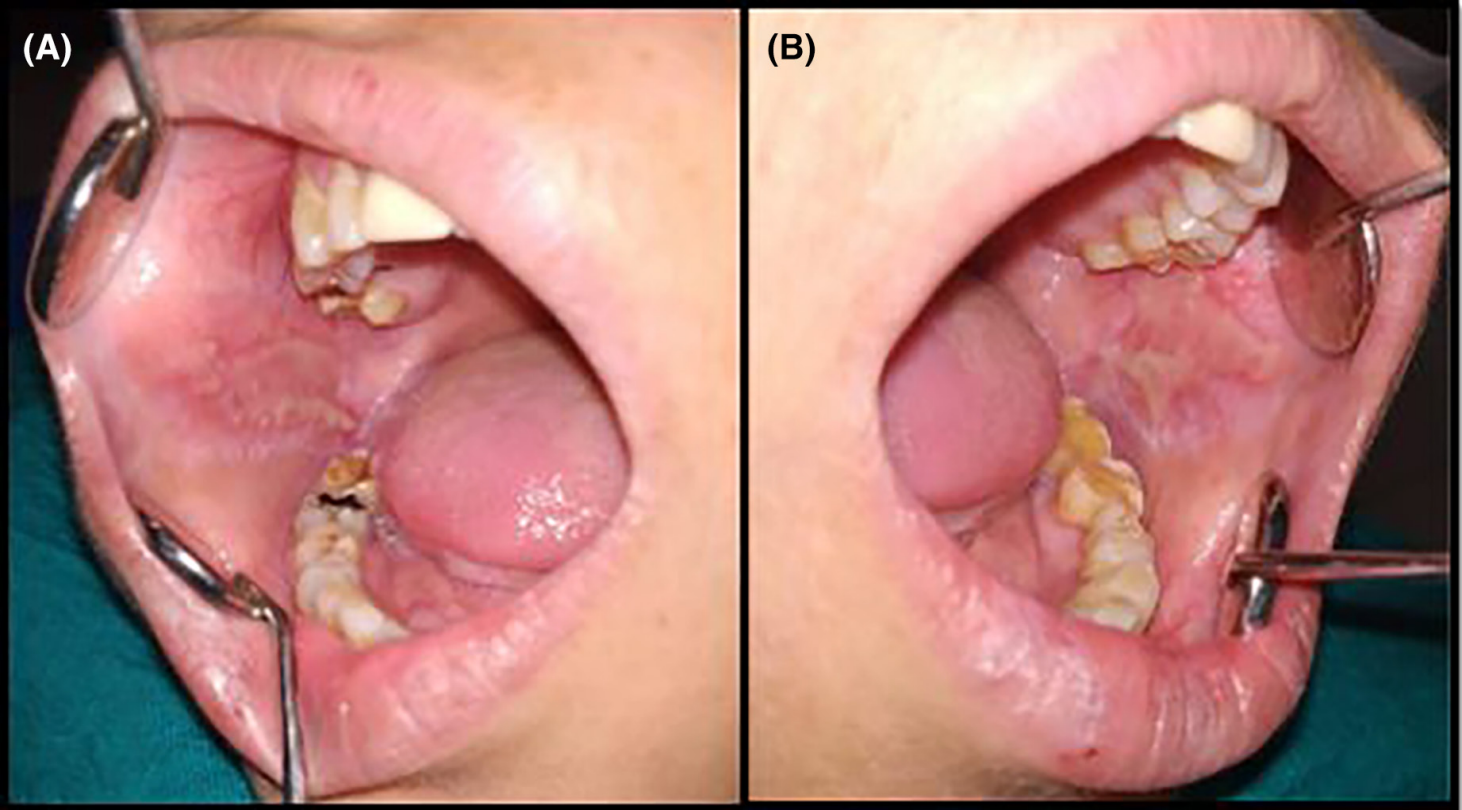
(A) Diffuse, irregular erosions on the right buccal mucosa. (B) Erosive lesion on the left buccal mucosa along the occlusal plane.

**FIGURE 2:**
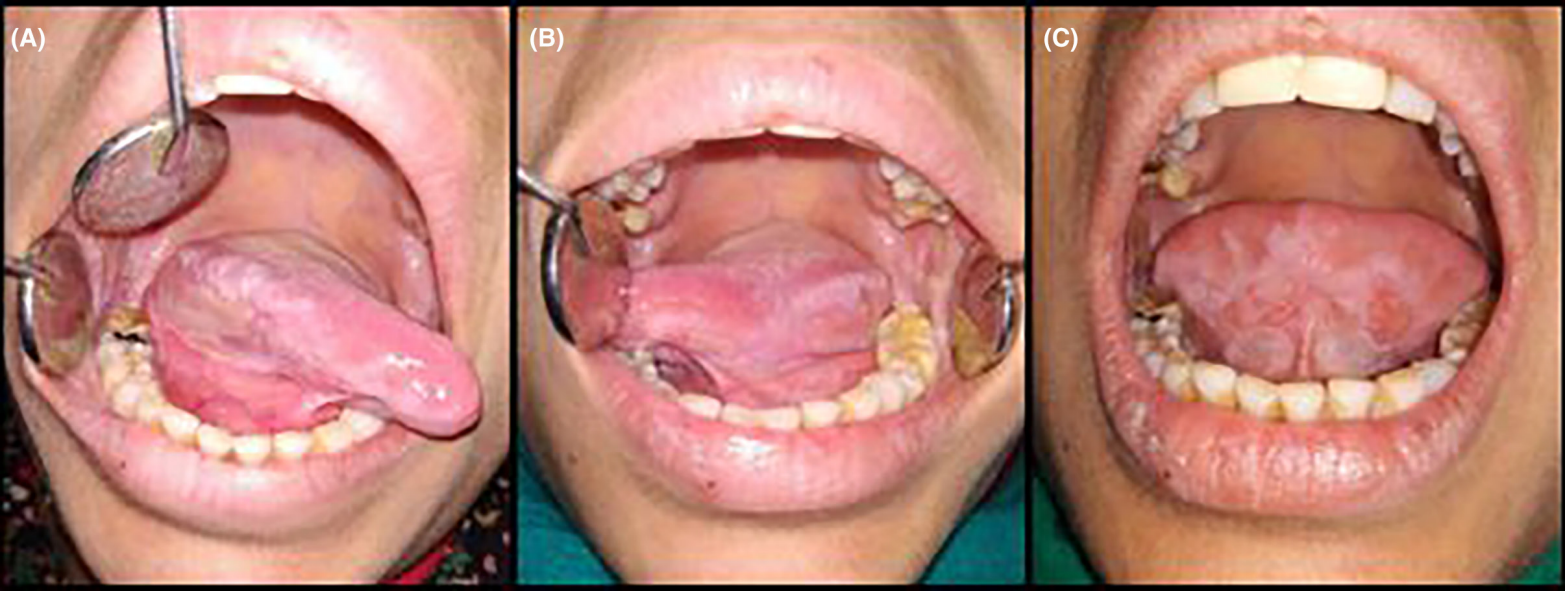
(A–C) Ragged erosions covered by fibrin slough on the tongue.

History revealed that the patient was apparently well 8 months before when she noticed bullae eruptions in the buccal mucosa that quickly ruptured to form erosive lesions. The erosions were initially painless but became painful over the last 6 months. The medical and family anamnesis was non‐contributory, and she denied the intake of any systemic medications. The patient consulted a few private practitioners and was treated conservatively with topical medications. However, the lesions did not respond to conservative therapy. The general physical examination was non‐contributory, with no cutaneous, conjunctival, or genital lesions. Intraoral examination revealed diffuse irregular erosive lesions extending bilaterally from the commissure to the retromolar regions on the buccal mucosa along the occlusal line. The erosions were covered with a pseudomembranous slough and surrounded by a mild keratotic zone (Figure 1A,B). Diffuse and ragged erosions were observed bilaterally on the lateral borders of the tongue. (Figure 2A,B). Erosions were also apparent on the ventral aspect of the tongue. (Figure 2C).

A positive Nikolsky sign I (application of mild lateral pressure on the apparently normal mucosa induces the formation of an erosive lesion) was elicited. The lesions were extremely tender and nonindurated on palpation. The patient reported poor oral hygiene and discomfort. Gingival inflammation with bleeding on probing, generalized attrition, and sharp cusps in the posterior mandibular teeth were also observed.

### 2.2 Differential diagnosis

Pemphigus vulgaris, mucus membrane pemphigoid, bullous lichen planus, herpetic gingivostomatitis, and erythema multiforme were placed in the list of differential diagnoses based on the history of multiple chronic erosions preceded by flaccid bullae formation and a positive Nikolsky sign.

### 2.3 Investigations

**FIGURE 3:**
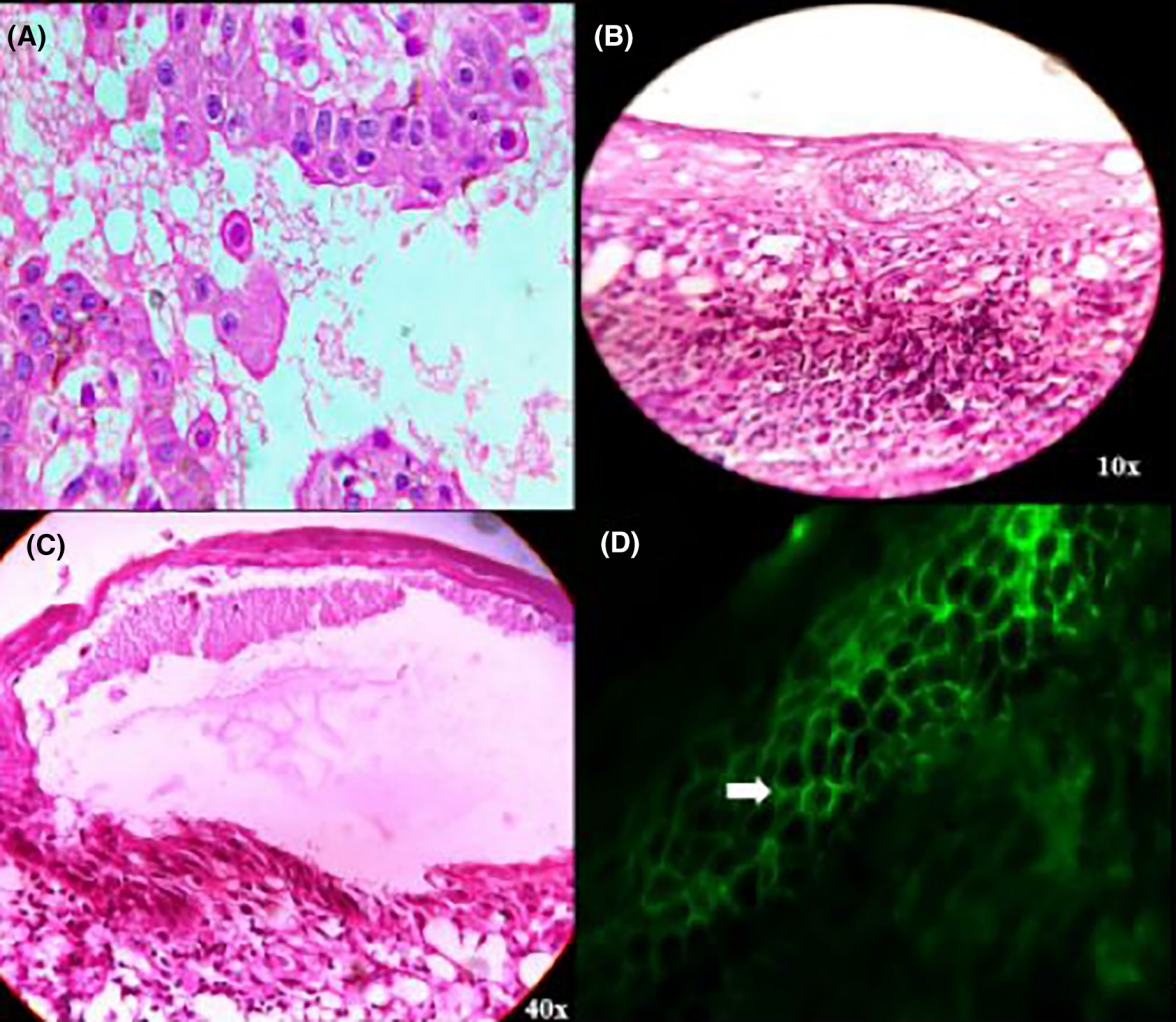
(A) Tzanck smear‐ reveals acantholytic cell. (B) Revealed a suprabasilar split and a tombstone pattern. (C) Showing the disrupted cellular junction projections towards the split. (D) Revealing the characteristic “chicken‐wire” or “fish‐net” appearance.

A smear was obtained from the lesion and stained with Hematoxylin and eosin (H & E). It revealed acantholytic cells with peripheral cytoplasmic condensation and large nucleus, while focal areas showed initiation of disruption in cell junctions (Figure 3A). An incisional biopsy including the perilesional tissue from the right buccal mucosa was obtained and submitted for routine histopathological examination and direct immunofluorescence (DIF) testing. Microscopic examination revealed a suprabasilar split in the epithelium, with loss of keratinocyte adhesion (acantholysis). The basilar keratinocytes are intact and form a tombstone pattern. Chronic inflammatory cells (predominantly lymphocytes) were observed in the split and juxta‐epithelial regions (Figure 3B). The sections also revealed basilar degeneration in focal areas with an intraepithelial cleft. The upper dermis showed chronic inflammation, along with the focal presence of eosinophils (Figure 3C). The stained section after DIF revealed classic “chicken‐wire” or “fish‐net” appearance. The white arrow shows intercellular junction staining of the stratified squamous epithelium with IgG (fluorescein isothiocyanate, ×200) (Figure 3D).

5 mL blood sample was collected from the patient and stored at −70°C. The Dsg titers were evaluated using enzyme‐linked immunosorbent assay (ELISA) with a kit from Medical and Biological Laboratories Co. Ltd., (Nagoya, Japan) following the manufacturer's instructions. A cut‐off value of >20U/mL was considered as positive. In the present case, Anti‐dsg 1 and anti‐dsg 3 values were 125.84 U/mL and 193.63 U/mL, thus confirming the diagnosis of Oral PV. A confirmatory PV diagnosis was made based on the characteristic cytological, histopathological, immunofluorescence features, and ELISA titers.

### 2.4 Treatment

The patient was prescribed a short course of topical steroids in the form of tablet betnesol 1 mg (swish and spit), turbocort oromucosal paste (0.1% w/w), and tablet Celin (vitamin C) three times daily for a week. Depura oral solution (60,000 IU vitamin D3) was also prescribed once a week for 4 weeks. Coronoplasty for sharp tooth cusps was performed and the patient received additional instructions regarding oral hygiene measures.

### 2.5 Outcome and follow‐up

**FIGURE 4:**
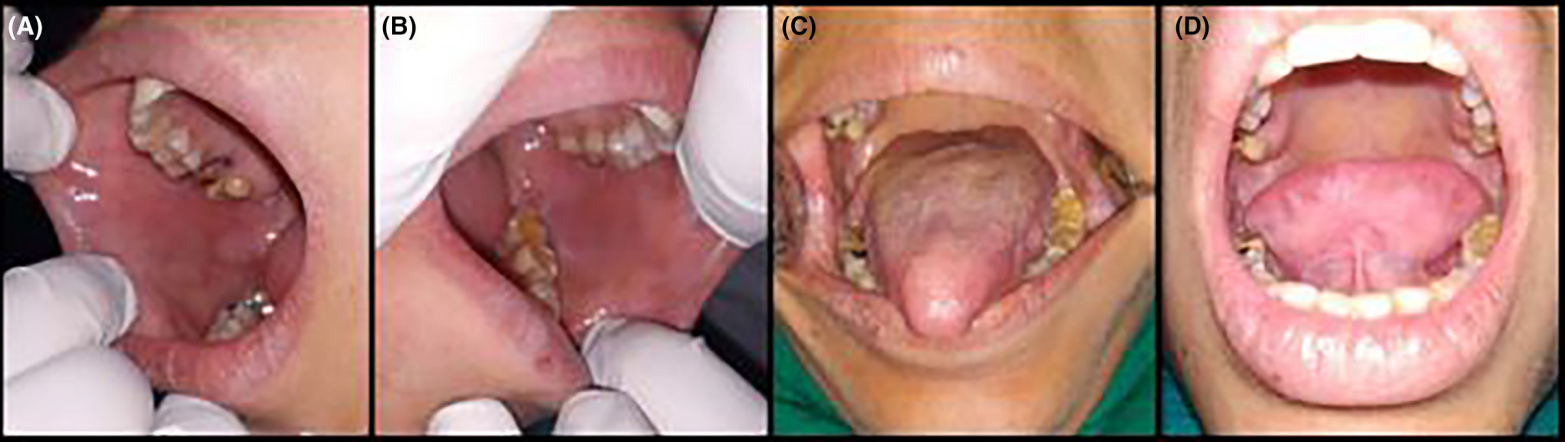
(A–D) Healed lesions.

The lesions showed complete resolution after 3 weeks of topical steroid therapy. The steroids were gradually tapered and discontinued (Figure 4A–D). The patient was regularly monitored for 1 year, and no recurrence was reported.

## 3 DISCUSSION

A comprehensive manual and electronic literature search was performed using PubMed and Google Scholar search engines employing following Medical Subject Headings (MeSH) terms, “Pemphigus Vulgaris”[Mesh]) AND (“Child”[Mesh] OR “Adolescent”[Mesh] OR “Pediatrics”[Mesh]. The articles published in English language between 2000 to November 2023 were included. Two authors methodologically assessed the titles and abstracts of the retrieved studies/case series/case reports, and any disparity was resolved by a third author. The references of all the included studies were manually checked to include any previously missed studies. A detailed literature research revealed only 53 cases of juvenile PV from 23 articles. The search approach exhibited the following attributes; age, sex, affected site, clinical features, diagnostic aids, treatment protocol, and follow‐up (Table 1).

**TABLE 1:** Summary of the reported juvenile pemphigus vulgaris cases.^10^, ^11^, ^12^, ^13^, ^14^, ^15^, ^16^, ^17^, ^18^, ^19^, ^20^, ^21^, ^22^, ^23^, ^24^, ^25^, ^26^, ^27^, ^28^, ^29^, ^30^, ^31^, ^32^

S.no.	Author (s) & year	No. of patient	Age/Sex	Clinical features	Mucosal involvement	Histopathology	IF	Therapy	Follow‐up/Relapse
1.	Pires et al.^11^ 2000	1	16/F	Erythematous, irregular ulcers on the skin of the back	Ulcers on lips, buccal mucosa, soft palate, tongue, and buccal gingiva	NA	Intercellular IgG and C3 on DIF	Systemic Pred 40 mg; gradually tapered to 5 mg/day.	Oral Pred (5 mg/day) maintenance therapy.
2.	Merchant et al.^12^ 2003	2	15/M	Throat ulcers with progressive lip, tongue, and buccal mucosal involvement. Profuse ocular watery discharge and photophobia, severe dysphagia	Erosions of the lips, gingiva, tongue, and buccal mucosa	Acantholysis with intraepidermal, suprabasal vesicle on HP	Intercellular IgG and C3 on IF. Positive anti‐desmoglein 3 serology on IIF	IV MP (1 mg/kg/d) followed by Pred 40 mg with calcium and vitamin D supplementation. Aza as an adjunctive therapy	3 months
			16/F	Throat ulcers with progressive lips, tongue, and buccal mucosal involvement	NA	Intraepidermal bulla, spongiosis, and acantholysis on HP	Intercellular IgG and C3 on DIF. Auto Ab titre 1:320	IV fluids and morphine for analgesia. IV methyl Pred (1 mg/kg/d) followed by Pred (2 mg/kg/d).	NA
3.	Ariyawardana et al.^13^ 2005	1	14/F	Multiple erosions and ulcers on lips, buccal mucosa, lateral borders of the tongue, soft palate and gingiva.	Oral mucosa	Acantholysis with intraepithelial blistering on HP	Intercellular IgG and C3 on DIF.	Systemic Pred 10 mg BD, tapered to 5 mg OD with dapsone 100 mg a day. Topical 0·1% TCA for residual lesions.	1 year follow up showed no relapse.
4.	Schmidt et al.^14^ 2005	1	14/M	Extensive erosions	Oral mucosa	NA	‐ IIF	Pred 80 mg qd, Aza 75 mg qd, Dap 75 mg qd initially with no improvement, switched to staphylococcal protein A immunoabsorption 57 cycles, followed by MMF 2 g qd, Cys 750 mg 3 pulses followed by MP 20 mg qd, MMF 1.5 g qd with no control, eventually switched to RTX qw, IVIG 2 cycles for 4 week.	4 years 6 months; Osteopenia, GIT candidiasis, bacterial superinfection of pemphigus lesions, Cushingoid appearance, conduct disorder at school, and growth retardation adverse effects
5.	Yazganoglu et al.^15^ 2006	3	13/M	NA	Oral, nasal mucosa	NA	Intracellular IgG, C3 deposition on DIF	MP (2 mg/kg/day) + Dap (50 mg/day)	2 years
			14/M	NA	Oral, ocular mucosa	NA	Intracellular IgG, C3 deposition on DIF	MP (1 mg/kg/day)	3 years
			15/M	NA	Oral mucosa	NA	Intracellular IgG, C3 deposition on DIF	MP (1.5 mg/kg/day) + MMF (2 g/day)	3 years
6.	Mamelak et al.^10^ 2007	2	16/F	Oral lesions heralded by pain and halitosis, followed by skin erosions in the head and neck region.	Oral mucosa	HP findings were diagnostic of PV.	+ IF findings	Refractory to I.M CRs initially, followed by oral Pred and MMF. Substantial improvement with oral Pred and Aza 200 mg daily.	Avascular hip necrosis due to systemic CR use. Acute flare up of oral & skin lesions treated with Pred 80 mg, Aza 200 mg, and IV IG. Remission was achieved after end of 15th day RTX 375 mg/m^2^ therapy. Azathioprine 200 mg daily as maintenance dose.
			16/F	Flaccid blisters, bullae, and desquamating epithelium over most of her body. Erosions on the buccal mucosa, gingiva, and hard palate	Oral mucosa	HP consistent with PV	+DIF	Skin lesions responded poorly with Pred 1 mg/kg and MMF 40 mg/kg daily. After 6 cycles of plasmapheresis completion, remission achieved with RTX 375 mg/m2 weekly and IVIG 2 g/kg.	MMF was discontinued and Aza 250 mg daily was started. Two additional doses of IV IG 6 and 10 weeks after her first infusion. Discharged on Aza 250 mg. The patient was treated with tapering Pred and monthly IV IG infusions after Aza therapy failure (secondary to elevated LFT).
7.	Asarch et al.^16^ 2009	8	15/F	NA	NA	NA	Auto Ab titre 1:160	I.V IG 2gm/kg/cycle over 3–5 days, every 3–4 weeks, increased to every 6,8,10,12,14,16 week	75 months
			14/F	NA	NA	NA	Auto Ab titre 1:320	I.V IG 2gm/kg/cycle over 3–5 days, every 3–4 weeks, increased to every 6,8,10,12,14,16 week	72 months
			15/M	NA	NA	NA	Auto Ab titre 1:80	I.V IG 2gm/kg/cycle over 3–5 days, every 3–4 weeks, increased to every 6,8,10,12,14,16 week	69 months
			16/F	NA	NA	NA	Auto Ab titre 1:80	I.V IG 2gm/kg/cycle over 3–5 days, every 3–4 weeks, increased to every 6,8,10,12,14,16 week	71 months
			15/F	NA	NA	NA	Auto Ab titre 1:160	I.V IG 2gm/kg/cycle over 3–5 days, every 3–4 weeks, increased to every 6,8,10,12,14,16 week	63 months
			16/F	NA	NA	NA	Auto Ab titre 1:320	I.V IG 2gm/kg/cycle over 3–5 days, every 3–4 weeks, increased to every 6,8,10,12,14,16 week	60 months
			15/F	NA	NA	NA	Auto Ab titre 1:1260	I.V IG 2gm/kg/cycle over 3–5 days, every 3–4 weeks, increased to every 6,8,10,12,14,16 week	92 months
			18/F	NA	NA	NA	Auto Ab titre 1:640	I.V IG 2gm/kg/cycle over 3–5 days, every 3–4 weeks, increased to every 6,8,10,12,14,16 week	NR
8.	Popadic et al.^17^ 2011	3	13/F	Flaccid blisters and erosions on trunk and extremities, erosions on the buccal and genital mucosa	Buccal mucosa and genital mucosa	TS + for PV	DIF + Auto Ab titre 1:640.	Pred (1 mg/kg/day) and Aza (2 mg/kg/day) with alendronate, calcium, and vitamin D	Prednisone tapered to 20 mg on alternate days, azathioprine 2 mg/kg/day.
			15/M	Erosions on buccal and genital mucosa	Buccal mucosa and genital mucosa	TS, HP features consistent for PV.	DIF + Auto Ab titre 1:40.	Initially, Pred (1 mg/kg/day) with calcium and vitamin D. After 4 months, Pred (0.3 mg/kg/day) and Aza (1.4 mg/kg/day). Four weeks later, Pred (0.3 mg/alternate day) and Aza (0.7 mg/kg/day).	18 months
			14/M	Erosions on the oral mucosa & trunk	Oral mucosa	TS, HP features for PV.	DIF + Auto Ab titre 1:640.	DM (100 mg) i.v. in 500 mL of 5% dextrose on 3 consecutive days, with a DM oral gel. Later, substituted with Dap (100 mg, once per day).	20 months
9.	Reguiai et al.^18^ 2012	1	14/F	NA	NA	NA	NA	Refractory to CR, IV IG, mood changes, acne, and gastric ulcer; treated with rituximab	62 month follow‐up, CR off therapy; disease relapse occurred
10.	Baratta et al.^19^ 2013	2	15/F	Oral blisters with bloody, purulent discharge	Oral & genital mucosa	HP features consistent for PV.	DIF, and ELISA + for PV	Oral Pred 60 mg daily with a topical CR, benzocaine, and MMF (20 mg/kg/day), eventually increased to 25 mg/kg/day.	16 months
			14/M	Erosions on scalp, oral cavity face, chest, and back	Oral mucosa	Acantholysis with suprabasal split on HP.	IIF +	Oral Pred at (40 mg once daily) with topical CRs tapered to 4 mg daily. MMF 500 mg twice daily, increasing to 1000 mg twice daily. 1–2 mg of prednisone as maintenance	16 months
11.	Chen H et al.^20^ 2013	1	17/M	Vesicles in oral cavity and lip, extremities, neck, trunk, groin, and buttocks	Oral mucosa	Acantholysis with suprabasal split on HP.	Intercellular IgG on DIF.	I.V MP (1.2 mg/kg/day) followed by intravenous RTX (500 mg)	Disease is under control without oral medications.
12.	Haberland et al.^21^ 2014	1	17/F	Gingival hypertrophy	Gingival mucosa	Inflammatory hyperplasia with acantholysis on HP.	Intercellular IgG and C3 on DIF. Auto Ab titre 1:80.	Surgical excision of hypertrophied gingival tissue	NA
13.	Vinay et al.^22^ 2014	5	17/M	NA	NA	NA	NA	Refractory to Aza, CR, DP, MMF, later treated with RTX	19 month follow‐up; Complete remission off therapy, however relapse seen post 8 month therapy. Died due to sepsis related complications
			17/M	NA	NA	NA	NA	Refractory to CR, and developed iatrogenic cushing; treated with RTX	18 month follow‐up; Complete remission on therapy, however relapse seen post 12 month therapy
			17/F	NA	NA	NA	NA	Refractory to CR; treated with RTX	17 month follow‐up; Complete remission off therapy, No relapse seen
			13/F	NA	NA	NA	NA	Refractory to Aza and CR, treated with RTX	14 month follow‐up; Complete remission off therapy, No relapse seen
			12/M	NA	NA	NA	NA	Refractory to Aza and CR, treated with RTX	12 month follow‐up; Control of disease activity, relapse seen after 11 months
14.	Srivastava et al.^23^ 2017	1	14/M	Multiple ulcers on buccal mucosa bilaterally, upper and lower labial mucosa, ventral, & lateral tongue	Labial and buccal mucosa, and tongue	NA	Photomicrograph showed immunoglobulin IgG accumulation in intracellular surface	Topical TCA 0.1% with topical analgesic and topical anesthetic. IV RTX 500 mg along with aloe vera gel.	The patient is on follow‐up.
15.	Salman et al.^24^ 2017	2	14/M	NR	Oral mucosa	NA	NA	MP for 32 months, Dap for a year, I.V IG 13 cycles, Aza 14 months, RTX 4 cycles.	Remission on rituximab and low dose Methyl prednisolone
			16/M	NR	Oral mucosa	NA	NA	MP for 28 months, Aza for 17 months, IV IG 10 cycles, MMF for 7 months, RTX 2 cycles.	Complete remission off treatment
16.	Gupta et al.^25^ 2017	2	12/M	NA	NA	NA	NA	Refractory to CR, and Aza, later treated with RTX	12 month follow‐up; Complete remission off treatment; No relapse
			12/M	NA	NA	NA	NA	Refractory to CR, and CYP, later treated with RTX	12 month follow‐up; Complete remission off treatment; No relapse
17.	Surya et al.^26^ 2018	1	12/M	Ulcers and erosions on the buccal & labial mucosa, ventral surface of tongue, and floor of mouth	Oral mucosa	Acantholytic cells on HP.	NA	Pred (20) mg OD in tapering dose, BM 1 mg TDS (squish and spit), and topical TCA for localized lesions	10 months without any recurrence of lesions.
18.	Hettiarachchi et al.^27^ 2018	1	15/M	Recurrent oral ulcerations	Bilateral buccal mucosa and the tongue	HP‐ intraepithelial blistering with acanthosis.	DIF revealed intercellular IgG deposits.	Tapering dose of Pred 10 mg for 1 week in conjunction with a mouthwash	Marked improvement. Further management of cutaneous lesions.
19.	Bilgic‐Temel et al.^28^ 2019	3	13/M	NA	NA	NA	NA	MMF (3 months), Aza (8 months), Dap for 2 months, MP (9 months), followed by RTX for 42 months.	Partial remission off therapy
			14/M	NA	NA	NA	NA	MP for 7 months and Aza for 2 months, MMF for 7 months, IV IG for 6 months, followed by RTX for 34 months.	Partial remission off therapy
			16/M	NA	NA	NA	NA	MP for 24 months and Aza for 12 months, followed by RTX for 9 months	Complete remission off therapy
20.	Broshtilova et al.^29^ 2019	1	14/F	NA	NA	NA	NA	Treated with RTX after being refractory to CR, DP therapy and developing adverse effects of CR	34 month follow‐up; CR; no relapse seen
21.	Sakhiya et al.^30^ 2020	1	17/M	NA	NA	NA	NA	Refractory to Pred and Aza; treated with RTX	Complete remission
22.	Kianfar et al.^31^ 2022	9	14/M	NA	NA	NA	NA	Refractory to Pred, Aza, MMF, CS; treated with RTX	NA
			14/F	NA	NA	NA	NA	Refractory to Pred, Aza; developed side effects due to CR therapy; treated with RTX	NA
			14/M	NA	NA	NA	NA	Refractory to concomitant Pred; treated with RTX	NA
			16/F	NA	NA	NA	NA	Refractory to concomitant Pred; treated with RTX	NA
			17/M	NA	NA	NA	NA	Refractory to concomitant Pred; developed dyspepsia; treated with RTX	NA
			16/F	NA	NA	NA	NA	Refractory to Pred, AZA; treated with RTX	NA
			16/F	NA	NA	NA	NA	Refractory to Pred, Aza, MMF; treated with RTX	NA
			17/F	NA	NA	NA	NA	Refractory to concomitant Pred; treated with RTX	NA
			16/F	NA	NA	NA	NA	Refractory to Pred; treated with RTX	NA
23.	Santiago‐Vázquez et al.^32^ 2022	1	14/F	Oral mucosa	Multiple scattered flaccid vesicles and erosive lesions	Lesional and perilesional skin biopsy	NA	Systemic Oral CRs (0.5 mg/kg/d), systemic steroids (MP 40 mg), IV IG therapy, followed by RTX therapy	Complete remission within 18‐month follow‐up; No relapse.

Abbreviations: Aza, azathioprine; BM, betamethasone; CR, corticosteroids; Cyp, cyclophosphamide; Cys, cyclosporine; Dap, dapsone; DIF, direct immunofluorescence; DM, dexamethasone; DMP, dexamethasone pulse; HP, histopathology; IIF, indirect immunofluorescence; IV IG, intravenous immunoglubulin G; MMF, mycophenolate mofetil; MP, methylprednisolone; NA, not available; Pred, prednisolone; PV, pemphigus vulgaris; RTX, rituximab; TCA, triamcinolone acetonide; TS, tzanck smear.

Juvenile/adolescent PV affects individuals between the ages of 12 and 18 years.^9^, ^22^, ^29^, ^33^ Our findings are in coherence with the published literature. Twenty seven males and 26 females were affected, and the youngest and oldest patients in our review were 12 and 17 years old respectively.

Chronic multiple oral erosions should always raise suspicion for pemphigus, even though erosions may recur in the early stages.^5^ As the oral mucosa may be the initial site of manifestation, dentists must consider PV in the differential diagnosis and eventually refer to further diagnosis and treatment.^34^ PV exhibits site predilection that commonly affects the skin and mucous membranes of the oral cavity, genitals, and conjunctiva. Pediatric PV is associated with a higher occurrence of genital and ocular lesions than adult PV.^33^ Moreover, the clinical progression of pediatric PV is often more unpredictable than that of adult PV,^2^ although it is generally considered to be better than that in adults.^19^

Our patient presented with exclusive erosive oral lesions at multiple sites. However, genital, ocular, and cutaneous examinations were non‐contributory.

The characteristic features of PV include flaccid intraepithelial blisters/bullae that eventually rupture and form painful ulcerations and erosions.^34^ A detailed history should emphasize the duration of symptoms, number of lesions, and frequency of recurrences.^26^ About 80–90% of patients develop oral lesions, which are often the first sign in approximately 60% of cases. These lesions often start as a blister/vesicle, and early lesions may appear as a solitary hemorrhagic bulla or shallow, irregular, or ill‐defined ulcerations.^5^

Vesicles/blisters, which eventually rupture to form erosions, are often observed at sites exposed to mechanical trauma or friction, such as the cheek mucosa, palate, lateral and ventral surfaces of the tongue, floor of the mouth, and lips.^5^

Intraoral lesions may exhibit a positive Nikolsky's sign due to perivascular edema that disintegrates dermal‐epidermal junction. However, smaller tense bullous lesions may also exhibit a positive Asboe‐Hansen sign/indirect Nikolsky sign/Nikolsky II.^7^ Patients with PV patients often complain of profuse salivation and difficulties with chewing and deglutition, as observed in the present case.^35^

Our patient presented with soreness and burning sensations in the oral cavity. Clinical examination revealed oral erosive lesions at multiple sites, with a positive Nikolsky sign. However, the cutaneous and other mucosal surfaces were not affected.

PV often poses a diagnostic threat if the oral cavity is the only affected site. The rarity of pemphigus lesions in pediatric patients further delays the diagnosis.^19^ PV should be given a place in the differential diagnosis of chronic erosive lesions of the oral cavity, and the oral physician should be familiar with the characteristic features of PV, thus, differentiating the disease from other erosive oral lesions.^19^

A comprehensive diagnosis of pemphigus is based on four criteria's‐ (a) clinical presentation, (b) histopathologic examination of a lesional biopsy, (c) direct immunofluorescence (DIF) examination of a perilesional skin or mucosal biopsy, and (d) Serological detection of autoantibodies against epithelial cell surface by indirect immunofluorescence (IIF) and/or enzyme‐linked immunosorbent assay (ELISA Dsg1 and Dsg3).^36^ Serological detection and differentiation of circulating autoantibodies by enzyme‐linked immunosorbent assays (ELISA) form the cornerstone of pemphigus diagnostics.^37^

Diagnostic tests for PV do not differ between adult patients and children, as both have similar clinical, histological, and immunological features. Cytodiagnosis can be a useful, cost‐effective, and quick diagnostic aid for ulcerative and vesiculobullous lesions, such as PV and herpes infections. Therefore, it can be used as a routine ancillary diagnostic method.^26^

In our case, smear revealed acantholysis and acantholytic/Tzanck cells with peripheral cytoplasmic condensation and a large nucleus.

Biopsies from intact vesicles/blisters yield more accurate results, but are infrequently seen as they tend to rupture easily.^26^, ^33^ Lesional skin samples were used for histopathological examination, and perilesional skin samples were used for direct immunofluorescence to detect tissue‐bound autoantibodies.^37^

Acantholysis and blister/vesicle formation within the epithelial layer, just above the basal layer (suprabasilar split), are the characteristic histological features of PV. In some cases, only the basal cells are left projecting into the blister cavity, creating a “row of tombstone” appearance.^1^, ^5^, ^7^ A relative scarcity of inflammatory cell infiltrates helps to distinguish PV from other bullous diseases, where profuse inflammatory cell infiltration is observed.^5^, ^7^

A suprabasilar split with acantholytic cells arranged in a tombstone pattern was observed in the present case, with features consistent with PV.

Serological investigations such as indirect immunofluorescence (IIF) and enzyme‐linked immunosorbent assay (ELISA) have also been conducted to detect autoantibodies against desmoglein glycoproteins.^35^ The quantitative ELISA titre can also be used to monitor the disease activity and response to treatment, as the antibody titre levels correlate with the disease activity and severity of the disease.^37^

The DIF findings indicate the presence of IgG antibodies that accumulate in the intercellular spaces, specifically targeting desmoglein proteins, resulting in a distinctive “fish‐net” or “chicken‐wire” appearance.^5^, ^7^ If the diagnosis remains unclear, immunoprecipitation and immunoblot analyses may also be performed.^35^

The primary objective of the therapeutic management of PV is to control the disease, heal mucocutaneous lesions, and minimize the associated functional disability. The therapeutic challenge is to prevent relapses and avoid adverse events associated with prolonged use of steroids and immunosuppressive agents. Thus, close clinical monitoring of the efficacy and safety of treatment is warranted.^38^

Systemic corticosteroids, either alone or in combination with adjuvant immunosuppressants, have traditionally been employed as treatment strategies for juvenile PV. Nevertheless, administering steroids alone may result in substantial adverse effects such as compromised physical appearance, increased susceptibility to infections, and nutritional deficits.^19^

Due to the limited number of controlled trials in pediatric PV, there are no approved therapeutic protocols by the Food and Drug Administration, and the existing therapeutic protocols lack substantial evidence. Currently, there are no specific guidelines for therapeutic strategies for this patient population.^19^, ^39^

Systemic corticosteroids form the cornerstone therapy for PV, while adjuvant therapies such as mycophenolate mofetil, azathioprine, dapsone, cyclophosphamide, and rituximab are used in recalcitrant cases. These current therapies are effective in reducing circulating antibodies and allowing patients to lead their normal lives.^40^ Prednisolone forms the cornerstone of treatment, and the appropriate dose in the pediatric group should be determined based on factors such as age, weight, severity of the disease, and potential side effects of the medication. The recommended dose of prednisolone is 0.5–1 mg/kg, which is gradually tapered to the lowest effective dose to minimize any associated adverse effects.^9^, ^19^, ^26^, ^34^ The steroid dosage may be modified based on the patient's clinical outcome, with gradual tapering in patients showing substantial resolution.^19^, ^33^ Oral PV cases may be successfully treated with a short course of low‐dose corticosteroids without the need for additional therapy, as was the case with our patient.^26^ However, long‐term steroid therapy is associated with adverse effects, such as obesity, growth retardation, osteoporosis, hormonal irregularities, and altered menstrual cycles. Therefore, alternative steroid‐sparing treatments and innovative therapeutic strategies to eradicate blisters at the molecular level are being actively sought.^40^

The prognosis of pediatric PV is generally better than that of adults; however, it may still be guarded owing to the potential adverse effects of corticosteroids and adjunctive immunosuppressants. Pediatric PV patients with extensive skin involvement (>70%), pneumonia, sepsis, and electrolyte imbalances are associated with mortality.^9^, ^26^

## 4 CONCLUSIONS

The rare occurrence of PV in the present case emphasizes the importance of considering PV as a possible differential diagnosis for vesiculobullous lesions in children and adolescents. PV may manifest with exclusive oral manifestations, and oral physicians should be familiar with the characteristic oral manifestations. A detailed history and clinical examination, coupled with histopathology and immunofluorescence, form the cornerstone of an early diagnosis and management.

## AUTHOR CONTRIBUTIONS

**Shyamkumar Sriram:** Conceptualization; writing – original draft; writing – review and editing. **Shamimul Hasan:** Conceptualization; methodology; supervision; writing – original draft; writing – review and editing. **Shahnaz Mansoori:** Data curation; investigation; supervision. **Shazina Saeed:** Investigation; methodology; writing – original draft; writing – review and editing. **Abhishek Banerjee:** Investigation. **Karthikeyan Ramalingam:** Investigation.

## FUNDING INFORMATION

This study received no external funding.

## CONFLICT OF INTEREST STATEMENT

The authors declare no conflicts of interest.

## CONSENT

Written informed consent was taken from the patient's parents for the publication of this report.

## Data Availability

The data that support the findings of this study are available from the corresponding author upon reasonable request.
